# Potential for Using Beetles (Coleoptera: Dermestidae) as Model Organisms to Determine Nutrient Bioavailability for Companion Animal Foods: A Pilot Study

**DOI:** 10.3390/ani15172630

**Published:** 2025-09-08

**Authors:** Mollie Toth, Charles G. Aldrich, Thomas W. Phillips

**Affiliations:** 1 Department of Entomology, Kansas State University, Manhattan, KS 66506, USA; 2 Department of Grain Science, Kansas State University, Manhattan, KS 66506, USA; aldrich4@ksu.edu; 3 Nulo Pet Foods, Austin, TX 78746, USA

**Keywords:** protein quality, PER, protein efficiency ratio, insects, Dermestidae, companion animal, pet food

## Abstract

Pet owners have become more aware of how their pet’s nutrition can influence the pet’s life expectancy, wellness, and energy. Evaluating the new ingredients claiming better pet health for nutrient contributions requires significant investment and targeted animal commitment while current research aims to find alternative approaches. Research on pet foods in universities is overseen by the Institutional Animal Care and Use Committee, which has restrictions on the use of vertebrates in testing but places no restrictions on the use of insects. The research reported here studied three species of beetles (Coleoptera: Dermestidae), *Trogoderma variabile* (Ballion), *Trogoderma inclusum* (LeConte), and *Dermestes maculatus* (DeGeer) as potential model organisms to evaluate pet food nutrient bioavailability. The larvae of all three species were fed a balanced pet food diet under various laboratory conditions to determine their protein efficiency ratios (PERs) over a 144 h period. Results suggested that *D. maculatus* larvae reared at 27 °C, 60–65% R.H. (relative humidity), and a photoperiod of 16:8 L:D were the most efficient at converting ingested protein into weight gain with an average protein efficiency ratio (PER) of 1.439. Further experiments were conducted using only *D. maculatus* larvae with shorter time windows of 48 h and 24 h. The 24 h feeding time was successful and yielded an average PER of 2.476. These findings suggest that *D. maculatus* larvae may be a useful model organism for pet food bioavailability studies.

## Simple Summary

Protein sources play a pivotal role in both consumerism and animal health in the pet food market. Current studies are highly restricted in using vertebrate animals as models. This study examined the plausibility of using three different insect species larvae, *Trogoderma variabile* (Ballion), *Trogoderma inclusum* (LeConte), and *Dermestes maculatus* (DeGeer), as model organisms for pet food bioavailability studies. The results were determined by measuring how well the insects were able to convert the protein of the diet into weight gain or a protein efficiency ratio. *Dermestes maculatus* larvae were observed to have the highest protein efficiency ratio value of 1.439 over a 114 h window. Additional experiments were carried out in 48 and 24 h windows with the 24 h window being most successful with a protein efficiency ratio of 2.476. With a higher protein efficiency ratio value over a lower time expenditure could imply that insects may be used as the new model organism for pet food studies by improving study efficiency.

## 1. Introduction

Proteins play a major role in companion animal nutrition and well-being. The amino acid composition can influence both the food’s digestibility and the proteins’ contribution to numerous body functions of the pet. For these reasons, many pet foods contain various protein sources to form a more complete profile, typically favoring animal sources [1,2]. In recent years pet food companies have been making substantial changes in their formulations due to consumer perceptions and concern about the purposes and health claims for various ingredients [3,4].

When evaluating various ingredients under consideration for inclusion in formulas, pet food companies can rely upon numerous techniques to determine protein quality [5]. Feeding studies in the target species can be expensive and time consuming, so rapid in vitro tests or evaluations with surrogates have been considered. Total protein content, amino acid composition, protein solubility, and in vitro digestibility techniques have been used, but they are typically incomplete in one or more aspects of proper evaluation [5,6]. Protein digestibility corrected amino acid scores add the layer of animal utilization, but rely upon a digestibility evaluation from a surrogate [7,8]. Another technique that can provide a macro evaluation of the protein ingredients quality is the protein efficiency ratio (PER) technique. This method determines the bioavailability of the first limiting amino acid in the protein source.

The first use of PER occurred over 100 years ago with albino rats (*Rattus norvegicus domestica* (Berkenhout)) in work by Osborne, Mendel, and Ferry (1919) to investigate the ability to compare various growth rates by limiting the growth of a negative control litter with protein-free milk, and soon the method became the standard of assessing protein quality [9]. Protein efficiency ratios allow for the various protein sources to be sorted based on their bioavailability of essential amino acids. PER studies using vertebrates all must conform to the laboratory regulations of the Institutional Animal Care and Use Committee in the USA (IACUC) [10], as well as to similar standards in other countries. To our knowledge, insects have not been used as research models to determine protein quality in foods.

Here we report a pilot study on the potential to use the feeding larval stages of three common stored product insects as models for estimating the PERs of commercial pet foods and as substitutes for vertebrate model organisms. Insect species included three different species of beetles (order Coleoptera) in the family Dermestidae: *Trogoderma variabile* (Ballion), *Trogoderma inclusum* (LeConte), and *Dermestes maculatus* (DeGeer). Dermestid beetles are a family of well-studied stored product pests. Stored product insects include hundreds of species of beetles, true bugs, booklice, and moths adapted to infesting durable foods produced by humans such as pet foods, stored grains, dried meats, and they are sometimes found on carrion [11,12,13]. These three dermestid species may have potential as invertebrate alternatives to vertebrates for determining PERs in pet foods because of their known dietary needs for high protein and fat levels as well as their behavioral habits of scavenging for carrion and other foods high in protein during their larval stages for growth.

Both *T. variabile* and *T. inclusum* are well-known economic pests and are found in various facilities including retail grocery and department stores, pet food stores, food processing facilities, and bulk storage structures [12,13,14]. These insects typically feed on various food sources including animal-based fabrics like wool and silk, pet foods, stored grains, and museum exhibits or old books with animal or grain derivatives. Both *Trogoderma* species cannot interbreed, but are morphologically very similar. The two species can be distinguished by discrete characters that require expertise to recognize, like the notching of the interior eye margin of *T. inclusum*, or genetic testing [15]. However, development and behaviors can sometimes be used for identification. *Trogoderma variabile* larvae develop slightly slower than *T. inclusum* larvae, taking approximately 34 days to reach full maturity, after which females can lay up to 90 eggs in their short week-long adult life span [16]. When their normal mating behaviors are disrupted, *T. inclusum* will typically lay eggs longer than *T. variabile*, leading to more eggs being laid and more progeny overall [17].

*Dermestes maculatus* is an economic pest that is less studied than the other two species being investigated. *Dermestes maculatus* is considered a pest of animal products including fish, pet foods, natural silk from the silk moth (*Bombyx mori*), mammalian fur, bird feathers, and related museum exhibits [18]. Their lifecycle differs in length with a variable number of instars (from 5 to 13) for development that are highly reliant on environmental factors including humidity, moisture content of their food source, and the type of nutrition provided [19]. Variation in development can cause larvae to take anywhere from 40 to 70 days to reach adulthood. Although not typical of dermestid beetles, adults of *D. maculatus* can survive for up to a year and a female can lay up to 800 eggs [20].

The ultimate goal of this work was to determine if insects could be used in place of vertebrate surrogate animals in bioavailability studies to forgo IACUC or similar regulations and create a faster process. In this pilot study it was essential to develop the parameters of the study with the alternate species. Insects are much easier to rear in the laboratory, can be used in much larger laboratory populations to provide more replicates than one would have with vertebrates, and have a substantially faster growth rate compared to the usual vertebrates used for PER experiments. Dietary bioassays were conducted with the larvae of *T. variabile*, *T. inclusum*, and *D. maculatus* to determine their PER values and feed conversion rates in a manner analogous to other PER model organisms reported in the scientific analysis of pet food studies. Given the foods being used as the base of evaluation would otherwise be considered complete from an amino acid perspective, it provided a substrate close to ideal. Based on the lifecycles described above, we predicted that *D. maculatus* would be the most efficient of the three dermestid species as a model organism for companion animal bioavailability studies. Variations in our initial experiment were conducted to account for unforeseen issues in the starting experiment.

## 2. Materials and Methods

### 2.1. Insect Colonies

Colonies of *T. variabile* and *T. inclusum* were acquired from the USDA ARS Center for Grain and Animal Health Research (CGAHR) in Manhattan, KS in 2017. Both *Trogoderma* species were reared separately inside 1 L glass Mason jars (Ball Co., Broomfield, CO, USA). Each jar contained a rearing diet that was approximately 250 g of ground Purina Complete Puppy Chow diet (Nestlé Purina PetCare Co., Ltd., St. Louis, MO, USA) with approximately 15 g of rolled oats (The Kroger Co., Ltd., Cincinnati, OH, USA) spread in a thin layer on top of the pet food. The sealed metal lid inserts of the glass jars were replaced with a fine-mesh metal screen to prevent beetle escape yet allowed for an exchange of fresh air and humidity. Crumpled paper towels were placed in the headspace of the colony jars to provide extra surface space for mobility of the insects. The colony of *D. maculatus* was also obtained from CGAHR and originated from wild-captured beetles infesting powdered blood at a pet food processing plant in 2009. *Dermestes maculatus* colonies were reared with approximately 325 g of ground Purina One Lamb and Rice diet (Nestlé Purina PetCare Co., Ltd.) spread into a 1 cm thick layer that was then thinly topped with approximately 35 g of rolled oats inside a clear 5.7 l. (30 cm × 15 cm × 9 cm) plastic box (Sterilite Co., Ltd., Townsend, MA, USA). Each box had a 3 cm diameter screened hole in the lid to allow for air flow while preventing insect escape. The rearing boxes also each had a 6.5 sq cm piece of “faux fur” fabric placed upside down near the end of the container as described by Fontenot et al. (2015) to promote egg laying, as well as two folded paper towels for shade and increased surface area for mobility [21]. A new colony of each species was started on a weekly basis. Each new colony was started with 60 mixed-sex young adults (1–14 days since emergence) placed in a colony box with fresh food, paper towels, and faux fur for one day after which all the adults were removed. This 24 h infestation assured that all progeny in a new colony box would be the same age within one day of each other.

Beetle colonies were maintained in a Percival I-66VL incubator (Percival Scientific Inc., Perry, IA, USA) at 27 °C with a 16:8 light–dark photoperiod and maintained at a relative humidity of ~60%. Two-week-old small larvae from each species, typically second or third instar after eclosion from the egg stage, were used in the bioassays described below.

### 2.2. Protein Content of Diets

Each experimental species group had twenty experimental units and ten pairs of two separate beetle larvae, each reared on different diets to measure weight gain or loss over a fixed time period. One diet was designated as a positive control (PC) and the other a negative control (NC), and PERs were calculated for each unit based on the difference in weight gain of each larvae. The PC was Purina One Lamb and Rice diet (PLR, Nestlé Purina PetCare Co., Ltd., St. Louis, MO, USA), available commercially, which was a complete and balanced diet for adult dogs with normal activity levels. The NC was composed of cornstarch and dextrose (added in a 2:1 percentage ratio), mineral premix (5.365%), soybean oil (5.365%), choline chloride (0.22%), and vitamin premix (0.203%), and was completely devoid of any added protein source. The NC diet was made from raw solid ingredients that were dehydrated and broken down into a fine powder.

Nitrogen typically constitutes approximately 16% of a protein’s structure and has a crude protein coefficient of 6.25 (100%/16% = 6.25) [22]. To measure the nitrogen content of the food, the crude protein coefficient of 6.25 was input into our nutrient composition instrument, a LECO FP928 Nitrogen and Protein Analyzer (LECO Co., Ltd., St. Joseph, MI, USA) that combusts the food to read the nitrogen content of the gases. Three samples of 3.0 g of both the PC and the NC diets were placed into the ceramic weigh boats designed for the machine, which were placed on the machine’s loader that deposited the sample for testing. The sample was then burned off at a temperature of 1100 °C, and a thermal conductivity cell was used to detect the nitrogen content of the burned-off gas to determine what percentage of the sample was protein. The protein percentages were then averaged from the three food samples and used in the calculations for the PER of the respective diet fed to the beetle larvae in a given experimental unit.

### 2.3. Effect of Protein Availability on Growth of Beetle Larvae

Single larvae were placed in small, ventilated glass vials with precisely weighed food. We used 15 mL (20 mm dia × 65 mm tall) glass shell vials (Thermo Fisher Scientific Inc., Waltham, MA, USA) for holding one larva with the experimental diet. We first weighed each empty vial individually without its snap-cap using an Accuris Instruments W3100A-210 scale (Accuris Instruments, Edison, NJ, USA). The vial weight was then tared before adding 0.5 g ± 0.005 g of the diet. Each larva was weighed on a Cahn 28 automatic electro-balance (C.Y. Scientific LLC, Irvine, CA, USA) and placed individually into one of the vials containing 0.5 g of either positive or negative control diets. Once all the vials were filled with diet and a single larva, they were capped with a ventilated lid and placed into each of the three test environments. The test environments were the countertop of a laboratory workbench (20 °C, 40% R.H.), the top rack of the incubator (27 °C, 60% R.H.), and the middle rack of the incubator (27 °C, 65% R.H.). Temperatures and RH were recorded at each of the three locations using HOBO’s (Honest Observer By Onset; Model UX100-003, ONSET Computer Corp., Bourne, MA, USA). The experiment took place in a laboratory space measuring 5.35 m × 5.51 m × 2.74 m. The countertop was 0.93 m off the floor and 1.82 m below the ceiling. Our incubator had a circulation fan in the top ceiling, giving more direct airflow and lower humidity to the vials located 39.37 cm below it on the top rack compared to vials placed 58.42 cm below the fan on the middle rack. After 6 days, individual larvae were weighed again with the larvae carefully removed and weighed individually with the respective glass shell vial and remaining food being weighed together.

This first experiment above suggested that *D. maculatus* would be a good model species to utilize in optimizing methods for insect-based PER data. A similar experiment was then performed with 30 PC vials, 10 in the top left of the incubator directly under the fan, 10 in the top right of the incubator away from direct fan aeration, and 10 on the middle rack of the incubator. There were 10 NC vials placed in the middle rack used to verify that the insects required adequate protein for insect growth. After 48 h, individual larvae were measured for any weight gain. The number of molting individuals halved from 70% in the first experiment to 35% in this second experiment.

Results from the secondary experiment showed promising, measurable PER values with a higher percentage of usable replicates (insects that had not molted). Another follow-up experiment with 20 PC vials and 20 NC vials placed in the middle rack of a Percival I-41VL incubator (Percival Scientific Inc., Perry, IA, USA) was set to 27 °C with a 16:8 light–dark photoperiod and maintained at a relative humidity of ~65% to aid in lowering the chances of molting. The middle rack of this incubator sat 76.2 cm below the circulation fan in the top ceiling. After 24 h, individual larvae were measured for any weight gain. By narrowing the feeding window to 24 h the molting rate was reduced to >2% giving the *D. maculatus* a high success rate in measurable PER values.

### 2.4. Data Analysis

The PER value for each larva was calculated as follows.

(1) $$\mathrm{PER}=\frac{\mathrm{larval}\;\mathrm{weight}\;\mathrm{gain}\;(g)}{\mathrm{protein}\;\mathrm{availability}\;\mathrm{for}\;\mathrm{absorption}}=\;\frac{\mathrm{final}\;\mathrm{larval}\;\mathrm{weight}-\mathrm{initial}\;\mathrm{larval}\;\mathrm{weight}}{[\left(\mathrm{initial}\;\mathrm{diet}\;\mathrm{weight}+\mathrm{vial}\;\mathrm{weight}\right)-(\mathrm{final}\;\mathrm{diet}\;\mathrm{weight}+\mathrm{vial}\;\mathrm{weight})]\;\times \;(\mathrm{Protein}\;\%\;\mathrm{decimal})}$$

Data collected were analyzed on each of the single variables measured (e.g., weight gain, protein intake, etc.) for the three insect species as a randomized complete block design using the General Linear Model procedure of Statistical Analysis Software OnDemand for Academics Studio (SAS Institute Inc., 100 SAS Campus Drive, Cary, NC, USA; 3.81 Enterprise Edition, 2022). When analyzing the interaction between both the incubator placement of the colonies and insect species, the data were analyzed as a Two-Way ANOVA using JMP Pro Software (SAS Institute Inc., version 16.0.0, 2021) and SAS. The insect species were considered the experimental unit and each was blocked in three environmental placements. The Bonferroni multiple comparison method for preplanned comparisons was used to control the fixed comparisons of differences between the experimental units of count data. Figures were graphed using GraphPad Prism Software (GraphPad, version 10.2.1, 2024).

## 3. Results

**Figure 1:**
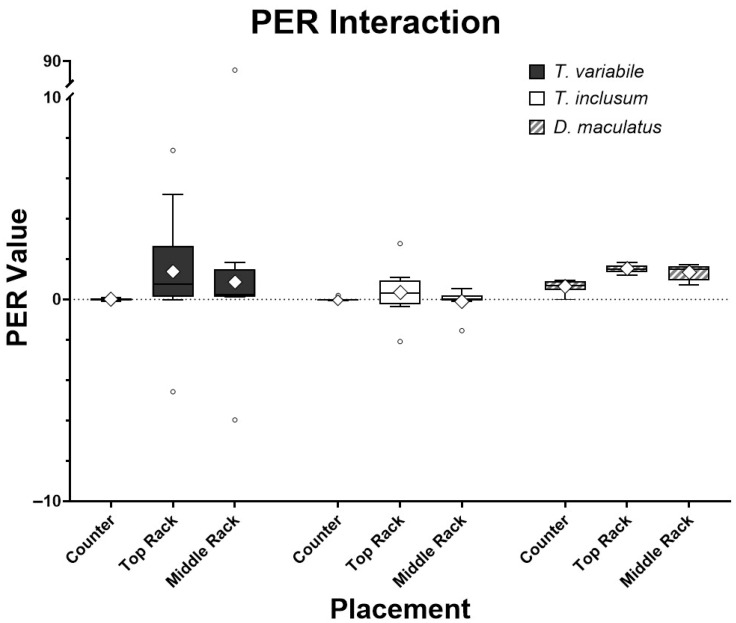
Comparisons among *T. variabile*, *T. inclusum*, and *D. maculatus* on protein efficiency ratios (PER) interaction in relation to bioassay locations: countertop of lab bench, top rack of incubator, or middle rack of incubator over a six-day period. Box-and-whisker plots show the standard mean (diamond), median (horizontal line through the box), box showing +/− 3 interquartile range, and whiskers showing minimum and maximum response with small dots for outliers. All treatments had ten individual larvae. The full model interaction has an F4,82 = 1.37 and *p*-value = 0.2522, analysis of the species alone has an F2,84 = 0.98 and *p*-value = 0.3783, and analysis of placement alone has an F2,84 = 0.96 and *p*-value = 0.3884.

The larvae from each of the three beetle species changed weight and consumed variable amounts of the diets in each environmental setting over six days (Table 1). When all the species were analyzed together and only the placements of the larvae were considered, the location played a significant role in their PER values (*p* < 0.05). However, when each species was analyzed separately the differences between the two Trogoderma species were not significant with respect to location while *D. maculatus* had significant differences in PER among locations, with a lower PER in insects reared on the counter and a higher PER when they were reared on the top rack of the incubator. Therefore, on an individual species basis, the location in the rearing chamber had no effect on *T. variabile* (*p* = 0.1969) and *T. inclusum* (*p* = 0.8853), whereas *D. maculatus* was affected by chamber location (*p* = 0.0003). When comparing the individual species to one another, the two Trogoderma species had significantly different (*p* < 0.05) PER values; however, there were no significant differences between PER values of Trogoderma species and *D. maculatus* (Figure 1, Table 1).

**Figure 2:**
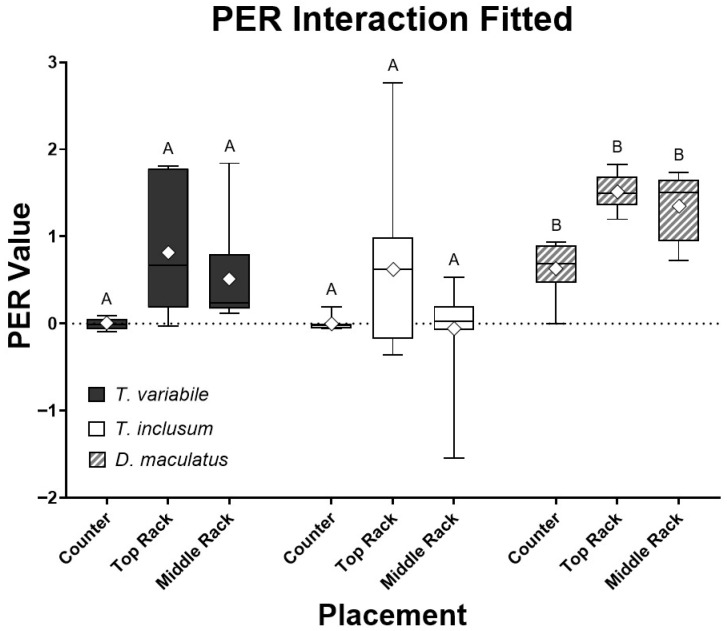
Comparison of all three species, *T. variabile*, *T. inclusum*, and *D. maculatus*, on protein efficiency ratio (PER) interaction in relation to bioassay locations (counter, top rack, or middle rack) over a six-day period using the standard mean, +/− 3 interquartile range after removing six major outliers. All treatments had ten individual larvae. The full model interaction has an F4,76 = 21.64 and *p*-value < 0.0001, analysis of the species alone has an F2,76 = 21.55 and *p*-value < 0.0001, and analysis of placement alone has an F2,76 = 10.5 and *p*-value = 0.0001. Note that none of the *Dermestes maculatus* data deviated from the results in Figure 1.

The PER values associated with insect species and place were compared together in an interaction analysis (Figure 1). There were four outliers for both *Trogoderma* species and none for *D. maculatus* when the PER values were analyzed using the interquartile range method [23]. *Dermestes maculatus* also had the smallest variation in PER values among all three species. However, the *p*-values of the full interactive model for species and placement were all non-significant (*p* > 0.05). Outliers were very likely caused by larvae molting while feeding and the diets absorbing moisture from the atmosphere, reflecting a lower ingestion rate or a higher weight gain, respectively, compared to others in that group. Larvae do not feed while molting followed by typical hardening and darkening of the exoskeleton, so there may actually be a reduction in weight gain compared to larvae that did not molt and consequentially show a low PER value. The water absorbed by the diets sometimes caused a reading of the replicates having more food present after the experiment than what was recorded before the experiment took place, increasing the PER value. Note that the major outlier in *T. variabile* on the middle rack, caused due to low food intake, lowered the denominator value, while molting to the next instar, increasing the numerator value and thus skewing the data. Once the major outliers were removed from the assay, species and placement interactions were significantly different (*p* < 0.001, Figure 2). On an individual basis, the only species that was significantly different in PER value was *D. maculatus* (*p* < 0.0001). All placements had significant differences (*p* < 0.05), with the PER values of insects reared on the counter and top rack of the incubator having the most significant difference (*p* < 0.001).

Results shown in Figure 1 and Figure 2 indicated that the ideal species of the three tested for use in further experiments was *D. maculatus*. Unlike both Trogoderma species, *D. maculatus* had reliably positive PER values eliminating the need to run an excessive number of replicates, since it is impossible to have a negative PER in ideal conditions with a subject eating a positive amount of food and gaining weight. Both Trogoderma species struggled with gaining weight compared to the rate of *D. maculatus* (Table 1). The PER results from *D. maculatus* had the qualities sought for in a PER model organism, with efficient conversion rates of energy intake to growth rates. However, an issue observed among all three species was larval molting, which needed to be addressed as it interfered with the anticipated weight gain recorded. The weight of the exoskeleton is not considered within the PER formula, and the larvae do not eat and are inactive during the molting process.

To address the problems associated with larval molting, two follow-up experiments were performed only using *D. maculatus*. In this experiment, adults were placed in a colony plastic box for three days to lay eggs. The two-week-old larvae were placed on either a positive control diet or an absence of diet for a shorter feeding period of 48 h in the incubator to determine if the time frame would be adequate to detect a weight gain. In all cases, larvae reared for 48 h had a decrease in overall molts with positive PER values. The data displayed variation in weight in the shorter time frame depending on whether insects were fed or not (Table 2). However, there were still too many molts in this tested 48 h time frame to effectively serve as a model for PER determinations.

The last experiment was performed with *D. maculatus* larvae placed on either a positive or a negative control diet for 24 h. The negative control diet was used for comparison this time because any time frame shorter than 24 h did not demonstrate enough weight difference to provide adequate PER readings. At 24 h of feeding, measurable PER values were obtained, and no molting was observed (Table 3).

## 4. Discussion

In this study, three dermestid beetle species, *T. variabile*, *T. inclusum*, and *D. maculatus*, were assessed for their ability to substitute as a PER model organism while fed different diets at different protein levels. To our knowledge, no previous studies have been reported using insects for PER bioassays. This dearth of reports and limited knowledge about their protein requirements or growth patterns led to multiple shortcomings in our procedures that required additional experimentation to refine the methods in this study. Such issues included (Table 1) factors such as the larvae that were in the incubator gained more weight in a six-day experiment than those on the counter. Ostensibly, this was due to the insects being ectothermic, and temperature has an impact on growth and reproduction. More specifically, these results were likely due to the insects in the incubator being able to ingest food with higher humidity (60% R.H.), which provided the hydration necessary to aid in digestion. Insects on the countertop may have lost water weight from the drier air (~40% R.H.) and used more energy for homeostasis, thus impacting their post-feeding weight. Another factor was assuring: that there was lower variation in data by having insects at the same life stage, and thus colonies were rotated on a weekly basis. An additional problem encountered was due to insect larvae molting during the experimental period, which created a loss in weight that was probably not due to diet intake. To counter the problems with molting, consideration of the temperature (whether it was inside an incubator or not) and the feeding timeline of the bioassay were addressed as potential issues. All the data collected through the three experiments suggested that *D. maculatus* was the best of the three Dermestidae species tested for purposes of this PER assay because of the consistency and higher weight gain, protein consumption, and PER values compared to those from the Trogoderma species. Thus, it can be concluded that out of the three species tested, *D. maculatus* has the highest likelihood of becoming a quality model organism in protein bioavailability studies to evaluate ingredients used in companion animal diets.

Prange et al. (1928) carried out similar experiments for growing chickens, which are still used today [24]. Other animal models for developing PER values of a food include pigs, dogs (usually beagles), and minks [5,7,25,26]. All these models have various benefits and have either similar or dissimilar dietary requirements to the target pets of interest, which can affect the values interpretation. As was determined in this study, external factors such as temperature and R.H. have the potential to influence PER values as well as the diets in question. The average PER values of this study using dermestid beetles ranged from −0.06 to 2.48, with *D. maculatus* ranging from 0.63 to 2.48. Other PER bioassay experiments using dogs, albino rats, and minks had PER values ranging from −2.99 to 5.32, 1.43 to 3.01, and 0.14 to 1.39, respectively [2,9,26]. These studies all show differing ranges based on multiple factors including the model organism of choice, ingredients of interest fed to the organisms, and potential external factors. Only one of these studies reported on the size of pens the model organisms were held in, while none reported on temperature or humidity, which all may have influenced the model organisms’ metabolism or feeding behavior. A benefit of using dermestid beetles would be that such external factor limitations may be minimized by utilizing environmental chambers. Proteins are essential to the nutrition and health of animals. Influencing structure and function down to the genetic level (nutrigenomics) has become critical in livestock applications, such as the influence on milk yield and fatty acid profile in muscle tissue which could eventually influence companion animal health research [27]. Although overlooked by many studies, proteins also provide nutrition to the gut’s microbiome after being digested by the host, which in turn can influence other health effects including inflammation, kidney disease, diabetes, heart disease, allergens, and oral health. The full scope of a protein’s influence on the animal’s health will likely never be fully understood or implemented within the pet food industry’s research. Continuing to find ways to further validate or test claims is vital in companion animal nutritional studies, including finding easier and more affordable ways to determine the bioavailability of protein sources. With the growing concerns of pet owners for their companions, many companies aim to aid in health and nutrition with direct claims on the front of their packages to reassure and inveigle consumers, often claiming higher quality and quantity of protein sources. Throughout the most recent years of the SARS-CoV-2 viral pandemic, and the years leading up to the pandemic, there were rising numbers of pet owners, people bonding with their pets, and a tendency to anthropomorphize their pets, leading to advances in the pet food industry [4,28]. Global pet food sales, predominantly for cats and dogs, have been increasing over the past decade with a nearly 70% increase from USD 78.1 billion to USD 114.8 billion from 2011 to 2021, respectively [29]. With an ever-growing market and an increasing clientele, pet food companies have been following trends in the human food industry to appeal to the owners, including vegetarian, vegan, and “premium diets” (which aim to target the owners’ lifestyles including non-GMO, sustainability, or natural ingredients); some of which claim to be healthier [29,30]. With owners growing a deeper connection to their pets they have been favoring brands that claim more health benefits and better nutrition compared to previous years, especially those claiming higher quality and quantity of protein. For these reasons, it is important to consider the costs, regulations, and management of the bioassays used for testing pet food ingredients, and use of insects in protein analyses could certainly satisfy these needs.

The evaluation of base diets in this study, which would otherwise be considered complete and balanced from a full nutritional perspective and (or) would be considered complete from an amino acid perspective, creates a platform to maximize the growth of the beetles and yield the highest potential PER value. Evaluating a complete diet is not the primary objective of the PER methodology, but rather to rank various protein ingredients that might be used in a complete food for their contribution of limiting and bioavailable amino acids. This assay has been used to determine impacts on thermal processing of proteins and rank proteins by various compositions (e.g., composition of hair, hide, hoof, wool, etc.). As a tool for protein ingredient evaluations having a model system that is relatively inexpensive, easy to manage, provides an alternative to vertebrate use and the liabilities associated with management (e.g., IRB review, IACUC, etc.) would be of great value. To that end, this pilot study appears to have provided a reasonable expectation for the use of dermestid beetles under certain growing conditions and time points to become a viable alternative. Several more evaluations will be necessary to verify this utility, such as comparison to vertebrate rankings of protein ingredient quality with that of the dermestid beetles in a side-by-side comparison. Further, continued optimization of time, temperature, and rearing practices will be necessary to completely define this model for use and incorporation into a widely accepted industry standard.

A limitation of this work is that we elected to not evaluate other known food storage insects and other insects at large. In short, this was a decision by the authors due to the prevalence of these beetles in and around high protein ingredients (e.g., rendered animal byproducts), which are common in pet foods, and due to their prevalence in and around stored pet foods [13,14]. It is clear because of this association that they are both attracted to and benefit from a nutritional perspective from these sorts of food items. In future work it would be valuable to consider other carnivorous species of insects in the event that they are more suitable for such research. Finally, the other limitation to this work is the cessation of feeding during an insect’s molting between larval stages, and the overall rapid lifecycle that is inherent with these species. Vertebrates and insects differ greatly in their rates of growth and they do not share, and may complicate, the very nature of the comparisons for studies of nutrient bioavailability.

## 5. Conclusions

This pilot study using insects as model organisms for pet food bioavailability studies may influence the pet food industry to reassess methods to analyze formulations. This study determined that *Dermestes maculatus* was the best of the three species tested and could be used for future bioavailability studies. Future experiments will need to refine the process further and test other variables. These experiments could include but are not limited to testing different insect species as models, humidities, temperatures, differing types of pet food, two-choice palatability bioassays, processing impacts, and amino acid analyses to determine their effects on potential insect PER values and make outcomes more replicable. If others validate insect testing for pet food quality, a major benefit to companies is to reduce the use of vertebrates such as mice, chickens, cats, or dogs as the preliminary screening tools to evaluate protein quality, lowering overall experimental costs, and producing fast results. These benefits could improve overall public perception of pet food processing and how the products are made.

## Figures and Tables

**Table 1:** Weight gain, food consumed, and calculated protein efficiency ratios (PERs) of three different beetle species in three different laboratory environments (n = 10 per row).

Insect Species	Location	Avg. (SE) Body Weight Gain (mg)	Avg. (SE) Diet Consumed (mg)	Avg. (SE) Gain–Feed Ratio	Avg. (SE) Crude Protein Intake (mg)	Avg. (SE) PER
*T. variabile*	Counter	−0.021 ± 0.04	10.28 ± 2.02	−0.002 ± 0.01	2.724 ± 0.54	−0.0063 ± 0.02
*T. variabile*	Incubator top rack	1.028 ± 0.29	3.71 ± 0.91	0.363 ± 0.27	0.983 ± 0.24	1.3684 ± 1.01
*T. variabile*	Incubator middle rack	0.847 ± 0.30	3.60 ± 1.08	2.871 ± 2.99	0.954 ± 0.29	10.8366 ± 11.28 **
*T. inclusum*	Counter	−0.014 ± 0.04	8.35 ± 0.69	−0.002 ± 0.01	2.213 ± 0.18	−0.0047 ± 0.02
*T. inclusum*	Incubator top rack	0.158 ± 0.08	2.93 ± 1.80	0.092 ± 0.11	0.776 ± 0.48	0.3464 ± 0.40
*T. inclusum*	Incubator middle rack	0.284 ± 0.11	15.31 ± 6.14 *	0.014 ± 0.05	4.058 ± 1.63	−0.0568 ± 0.18
*D. maculatus*	Counter	2.985 ± 0.51	16.99 ± 1.18	0.167 ± 0.03	4.502 ± 0.31	0.6313 ± 0.09
*D. maculatus*	Incubator top rack	8.791 ± 0.79	22.51 ± 2.53	0.401 ± 0.02	5.965 ± 0.67	1.5130 ± 0.07
*D. maculatus*	Incubator middle rack	7.969 ± 1.27	22.59 ± 2.42	0.350 ± 0.03	5.985 ± 0.64	1.3490 ± 0.13

* This calculation includes one major outlier with 53.1 mg of diet consumed. ** This calculation includes one major outlier with PER of 89.591.

**Table 2:** The growth performance and protein efficiency ratios (PERs) of *Dermestes maculatus* in three different environments (n = 10 per diet/location combination) for 48 h. Any of the larvae that had molted were discarded from the experiment.

Diet	Location	Number of Used Values	Avg. (SE) Body Weight Gain (mg)	Avg. (SE) Diet Consumed (mg)	Avg. (SE) Gain–Feed Ratio	Avg. (SE) Crude Protein Intake (mg)	Avg. (SE) PER
Purina One Lamb and Rice	Top left rack	7	3.085 ± 0.46	6.243 ± 0.66	0.466 ± 0.05	1.654 ± 0.18	1.7588 ± 0.20
Purina One Lamb and Rice	Top right rack	8	3.336 ± 0.81	5.55 ± 0.75	0.531 ± 0.13	1.471 ± 0.20	2.0042 ± 0.48
Purina One Lamb and Rice	Middle rack	3	2.709 ± 0.35	4.967 ± 0.87	0.557 ± 0.04	1.316 ± 0.23	2.1004 ± 0.13
None	Middle rack	8	−0.4671 ± 0.05	0 ± 0.00	-	-	-

**Table 3:** The growth performance and protein efficiency ratio (PER) of *Dermestes maculatus* in the middle rack of the incubator for 24 h (n = 20 per diet).

Diet	Avg. (SE) Body Weight Gain (mg)	Avg. (SE) Diet Consumed (mg)	Avg. (SE) Gain–Feed Ratio	Avg. (SE) Crude Protein Intake (mg)	Avg. (SE) PER
Purina One Lamb and Rice	1.572 ± 0.13	2.36 ± 0.19	0.656 ± 0.03	0.625 ± 0.05	2.4759 ± 0.13
Negative Control	0.142 ± 0.02	−1.105 ± 0.08	−0.163 ± 0.04	0 ± 0.00	-

## Data Availability

The raw data supporting the conclusions of this article will be made available by the authors on request.

## Abbreviations

The following abbreviations are used in this manuscript:

PER	Protein efficiency ratio
R.H.	Relative humidity
IACUC	Institutional Animal Care and Use Committee
CGAHR	Center for Grain and Animal Health Research
PC	Positive control
NC	Negative control
PLR	Purina Lamb and Rice
